# Single-Cell Transcriptome Analyses of Four Pain Related Genes in Osteosarcoma

**DOI:** 10.1177/11769351251331508

**Published:** 2025-07-19

**Authors:** Mesalie Feleke, Haiyingjie Lin, Yun Liu, Liang Mo, Emel Rothzerg, Dezhi Song, Jinmin Zhao, Wenyu Feng, Jiake Xu

**Affiliations:** 1 School of Biomedical Sciences, The University of Western Australia, Perth, WA, Australia; 2 Department of Orthopaedics, The First Affiliated Hospital of Guangxi Medical University, Nanning, Guangxi, China; 3 Shenzhen University of Advanced Technology, Guangdong, China; 4 Department of Orthopaedics, The Second Affiliated Hospital of Guangxi Medical University, Nanning, Guangxi, China

**Keywords:** osteosarcoma, scRNA sequencing, NGS, bioinformatics, osteoblastic cells, sarcoma

## Abstract

**Objective:**

Osteosarcoma (OS) is a rare and complex form of cancer that mostly affects children and adolescents. Pain is a common symptom for patients in OS which causes significant unhappiness and persistent aches. To date, there is minimal knowledge on the mechanisms underlying OS induced pain and few treatment options for patients. Previous genetic studies have demonstrated that the panel of four genes, artemin (*ARTN*), persephin (*PSPN*), glial cell line-derived neurotropic factor (*GDNF*), and neurturin (*NRTN*) are associated with the regulation of pain processing in OS and analgesic responses.

**Methods:**

In the present study, by utilising a scRNA-seq OS dataset, we aimed to measure the gene expression levels of four pain related genes, and compare them between the different cell types in human OS tissues and cell lines.

**Results:**

Within a complex and diverse range of cell types in OS tissues, including osteoblastic OS cells, carcinoma associated fibroblasts (CAFs), B cells, myeloid cells 1, myeloid cells 2, NK/T cells, plasmocytes, *ARTN* and *NRTN* genes had the highest expression in Osteoblastic OS cells, *GDNF* gene had a peak expression in carcinoma associated fibroblasts, and *PSPN* gene in endothelial cells. In addition, all four genes showed deferential pattern of expression in 16 OS cell lines.

**Conclusion:**

Future studies should investigate the potential to target deferentially expressed pain-related genes in specific cell types of OS for therapeutic benefit to improve the quality of life for patients living with pain caused by OS.

## Introduction

Osteosarcoma (OS) also known as osteogenic sarcoma is a type of cancer that originates in osteoblast cells that form bone. OS can spread to distant sites in the body such as the lungs increasing chances of death.^1^ It occurs primarily in children and young adults with an initial peak in the years 10 to 14 correlating with the pubertal growth spurt.^2^ Blacks are the ethnic group most affected by OS followed by Hispanics and Whites, respectively.^2^ The incidence of OS is higher in males than in females with an incidence of 5.4 cases per year per million and four cases per year per million, respectively.^2^ In terms of its pathophysiology, OS most frequently occurs approximate to the metaphysis of long bones in the skeleton followed by the femur, tibia, and humerus,^2^ and is divided into seven subtypes, including conventional OS (osteoblastic, chondroblastic, fibroblastic), telangiectatic OS, low-grade OS, small-cell OS, parosteal OS, periosteal OS, and high-grade surface OS.^1^ The current treatment options for patients include surgery, chemotherapy, samarium, targeted therapy, and radiotherapy that are dependent on position, size and stage of the of the tumour. The five-year survival rate for OS is approximately 70%.^1^ The quality of life of OS patients has improved over the last several decades.^2^ However, our knowledge of the aetiology of OS is obscure. Besides, our understanding of the cellular and molecular mechanisms underlying OS pathogenic pain remains minimal.

ScRNA-seq is a recently developed genomic technique that allows researchers to measure the gene expression of each individual single cell in a tissue.^3^ Previous scRNA-seq and bioinformatic studies by our group have shown that OS is highly heterogenous across patients and within the same tumour.^4,5^ Other labs have identified candidate genes involved in the development and progression of OS including mutations in tumour protein (TP53), Kirsten rat sarcoma virus (Kras), MYC, and retinoblastoma (Rb).^6-8^ However, the expression of pain related genes have not been well elucidated in human OS tissues and cell lines.

Pain could be a dismal and emotional experience and is the most common symptom of OS metastases.^9,10^ The development of OS induced pain is complex and the mechanisms that maintain it are unclear.^11^ For example, in a previous study, 91.6% of patients with metastatic bone disease experienced pain, with 36.2% reporting intense pain.^12^ In the present study, we have sought to examine the expression of four genes that are associated with the regulation of pain processing and analgesic response in OS and other pathological conditions, including artemin (*ARTN*), persephin (*PSPN*), glial cell line-derived neurotropic factor (*GDNF*), and neurturin (*NRTN*).^13,14^ It is aimed that this study will improve our understanding of the pathological pain in OS.

## Methods

### GEO Database Analyses

Gene expression profile were compared using the GEO database with the dataset GSE42352, which consists of genome-wide gene expression profiling of different sources, including mesenchymal stem cells (MSCs), osteoblasts, OS cell lines, and OS biopsy.^15,16^ As previously described, 113 sets of normalised gene expression data of the OS biopsies and OS cell lines were defined as the tumour group. Fifteen sets of normalised gene expression data of MSCs and osteoblasts were defined as the normal group.^17^ Student’s *t*-test, was performed and a *P* value < 2.2 × 10^−16^ value is considered statistically significant.

### ScRNA-seq Data Collection

The OS scRNA-seq datasets were retrieved from previously published datasets. We analysed the GSE162454 datasets (available at https://www.ncbi.nlm.nih.gov/geo/query/acc.cgi?acc=GSE162454).^18^ This dataset comprises 29 278 cells isolated from six primary tumour samples of OS patients. We proceeded by analysing four pain related genes in the single cells for subsequent analysis.

### Cell Clustering and Differential Expression Analysis in OS Cells

We conducted cell clustering and visualisation using Uniform Manifold Approximation and Projection (UMAP) with the Dimplot function, setting the parameters to ‘dim=1:30’ and ‘resolution=0.10’. Colour adjustments were made using the ‘ggsci’ package (version 2.9). The scale was done according to the Seurat package process scale of data (https://satijalab.org/seurat/articles/pbmc3k_tutorial). The cell type definitions aligned with those from our earlier studies.^5,18^ Subsequently, we used the FeaturePlot function to display gene expression distribution. Expression level was measured in transcript per million (TPM).

### Cell Quality Control

Data quality control was conducted using the same parameters from the Seurat package (version 3.2.1) as outlined in the author’s previous study.^18^ In brief, to ensure high data quality, cells were filtered out if they had between 300 and 4500 expressed genes and a mitochondrial gene percentage greater than 10%. After this filtering process, 29 278 cells remained for the identification of differentially expressed genes (DEGs). Kruskal-Wallis test was performed and a *P* value < 2.2 × 10^−16^ value was considered statistically significant. Finally, the Harmony package (version 1.0; https://github.com/immunogenomics/Harmony) was utilised to integrate the data.

### Cancer Cell Line Encyclopedia (CCLE) Dataset Analyses

Cancer cell lines are the most commonly used models for studying cancer biology, validating cancer targets and for defining drug efficacy.^19^ Therefore, cancer Cell Line Encyclopedia (CCLE) dataset was used to obtain related gene expression in OS cell lines. The CCLE database has performed large-scale deep sequencing on 947 human cancer cell lines derived from more than 30 different tissue sources, integrating genetic information including DNA mutations, gene expression, and chromosomal copy numbers.^19^

### Kaplan-Meier Survival Analysis

Kaplan-Meier survival analysis was carried out by using R’s survival packages as described previously.^17^ K–M < .05 was used as a cut-off criterion for the indication of a survival-related gene.

## Results

### Differential Expression of ARTN, GDNF, NRTN, and PSPN Based on the Human OS Dataset GSE42352

**Figure 1.**
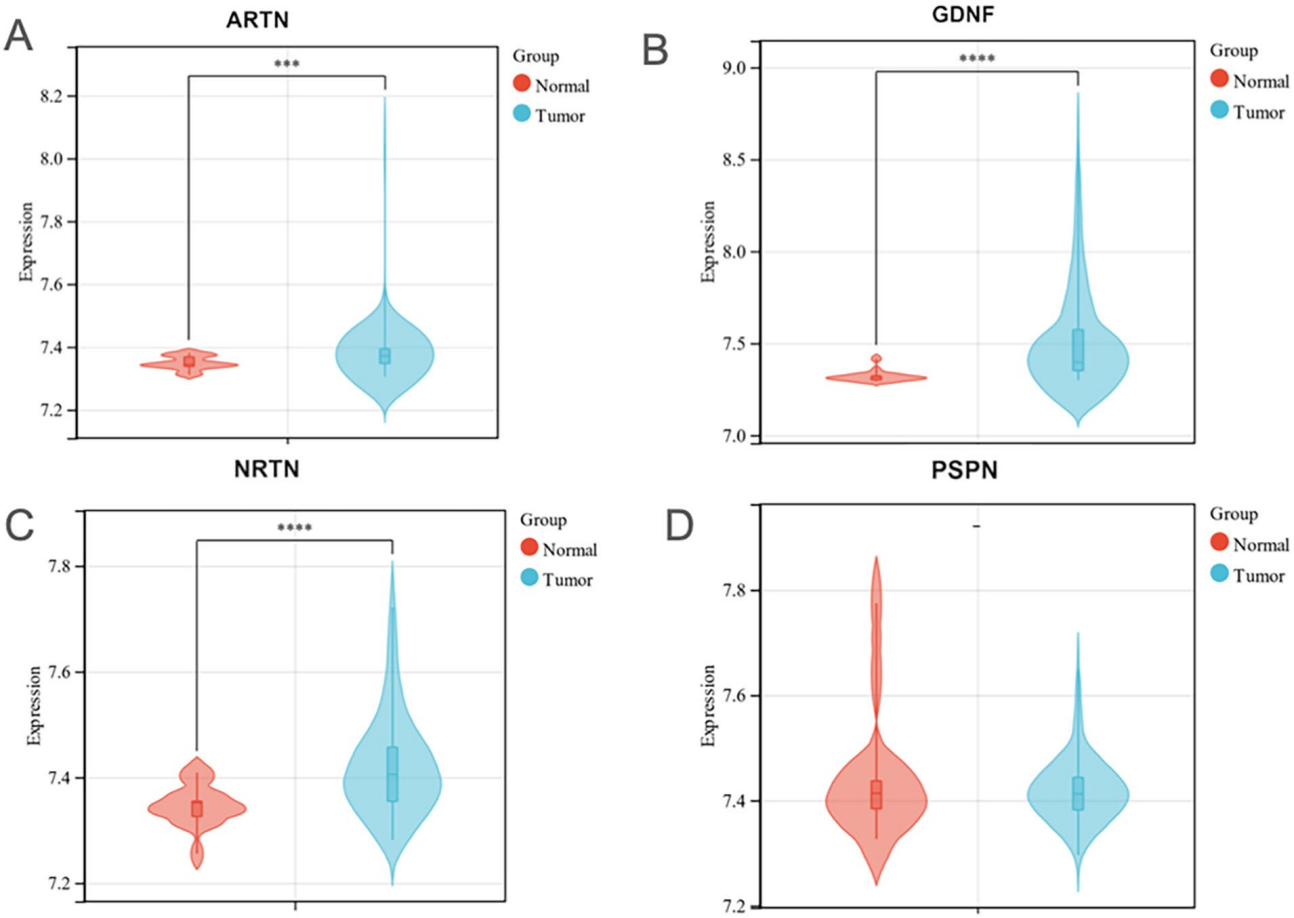
Violin plots showing the gene expression levels of *ARTN (A), GDNF (B), NRTN (C), and PSPN (D)* in OS and normal tissues. The violin plots filling in red representing normal groups, and the blue plots represent OS groups. The *y*-axis indicates the expression level of the genes. By using Student’s *t*-test, *P* < .05 was considered statistically significant (***P < .001, ****P < .00001).

To compare the gene expression profile of *ARTN, GDNF, NRTN, and PSPN* between *OS* samples versus normal tissues, the gene expression plots were generated using the dataset GSE42352. The results showed that the *ARTN, GDNF, and NRTN* transcripts were found to be upregulated in human OS cells when compared with the control (Figure 1), whereas the expression of *PSPN* show no difference between human OS cells and the control.

### Cellular Landscape of the OS Tumour Cell Microenvironment

**Figure 2.**
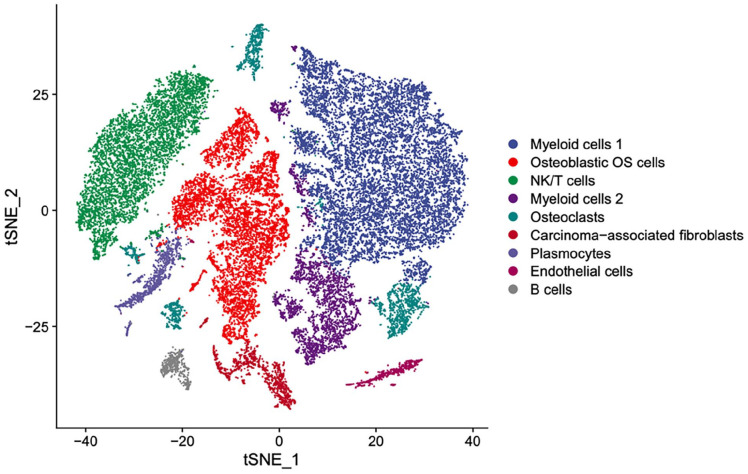
Uniform Manifold Approximation and Projection (UMAP) depiction of scRNA-seq data showing the 9 main cell types in OS.

Our scRNA-seq obtained from results indicate that the cellular composition of OS includes myeloid cells 1, osteoblastic OS cells, NK/T cells, myeloid cells 2, osteoclasts, carcinoma-associated fibroblasts, plasmocytes, endothelial cells, and B cells (Figure 2).

### ARTN, GDNF, NRTN, and PSPN Gene Expression in Osteoblastic OS Cells Versus All Other OS Cells

**Figure 3.**
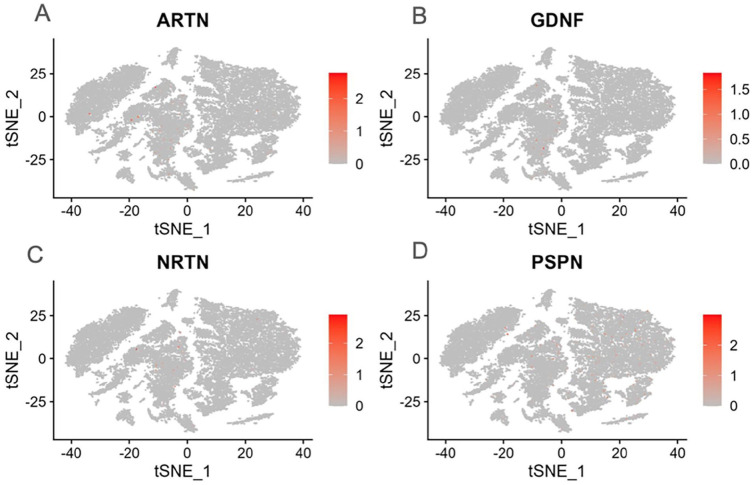
Uniform Manifold Approximation and Projection (UMAP) plot showing the relative mRNA expression in TPM of *ARTN (A), GDNF (B)*, *NRTN (C)*, and *PSPN (D)* in the 9 main OS cell types.

**Figure 4.**
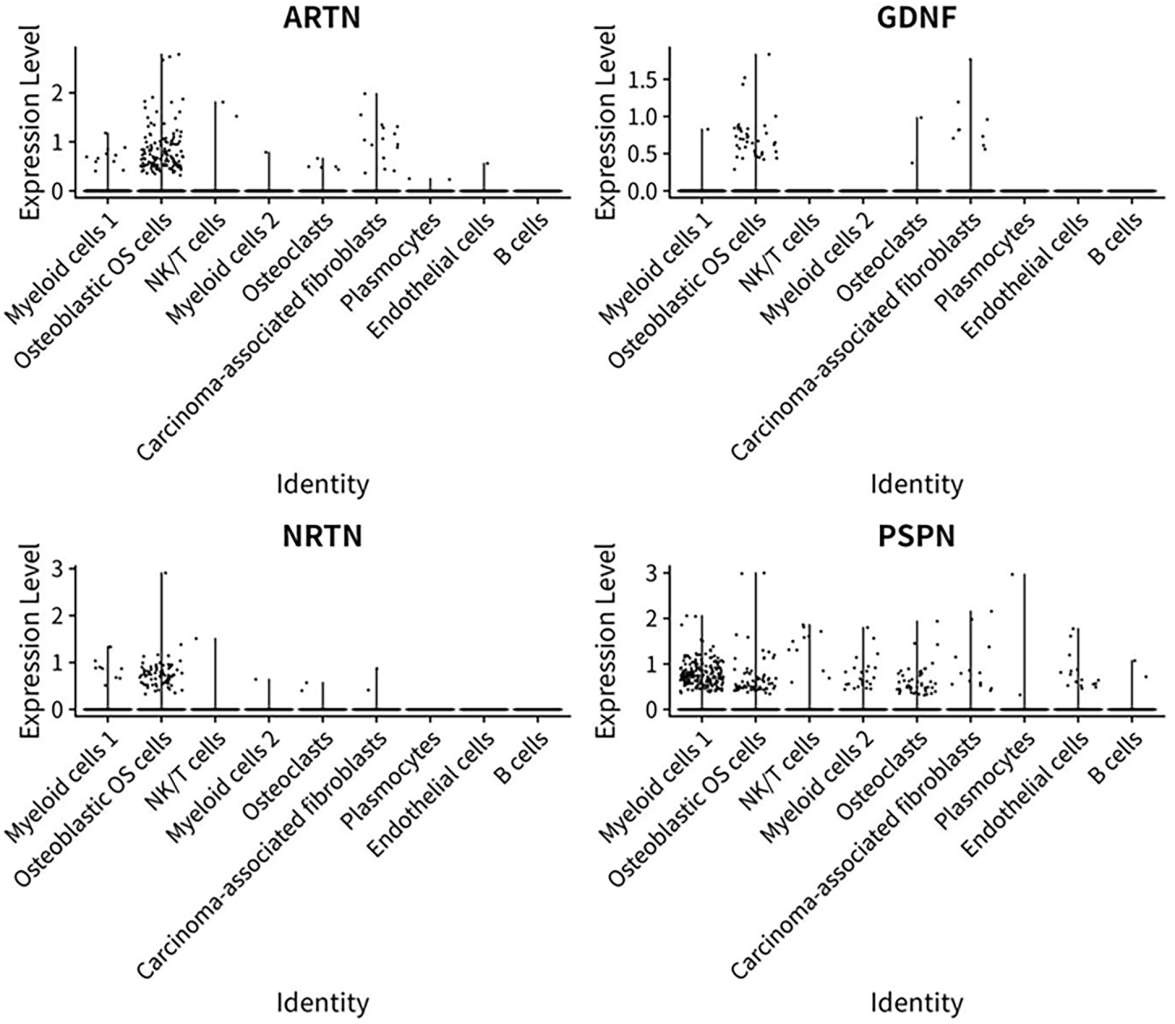
Dot plot displaying expression levels in TPM of four pain related genes in diverse OS cells.

The *ARTN* gene was highly expressed in osteoblastic OS cells (Figures 3 and 4). It was observed that the ARTN gene exhibited significant differential expression (DE) between Osteoblastic OS cells and NK/T cells (*P*-value = 1.816 × 10^−24^). However, the *ARTN* gene did not show DE between osteoblastic OS cells and myeloid cells 2 (*P*-value = 2.43 × 10^−13^) and osteoblastic OS cells and plasmocytes (7.58 × 10^−4^). Additionally, there was no significant DE of the *ARTN* gene between osteoblastic OS cells and osteoclasts (*P*-value = 1.37 × 10^−6^) and Osteoblastic OS cells and endothelial cells (*P*-value= 2.76 × 10^−2^). Furthermore, the *ARTN* gene was not DE between osteoblastic OS cells and carcinoma-associated fibroblasts (*P*-value = 1). The *ARTN* gene was not DE between B cells and osteoblastic OS cells (*P*-value = 2.76 × 10^−2^).

Overall, our findings showed that the osteoblastic OS cells had low expression of *GDNF* (Figure 4). It was revealed that the *GDNF* gene exhibited no significant DE between osteoblastic OS cells and NK/T cells (*P*-value = 4.32 × 10^−8^). Moreover, the *GDNF* gene did not show DE between osteoblastic OS cells and myeloid cells 2 (*P*-value = 2.27 × 10^−4^) and osteoblastic OS cells and plasmocytes (1.7 × 10^−4^). Additionally, there was no significant DE of the *GDNF* gene between osteoblastic OS cells and osteoclasts (*P*-value = 7.39 × 10^2^) and osteoblastic OS cells and endothelial cells (*P*-value = 6.01 × 10^−1^). Furthermore, the *GDNF* gene was not DE between osteoblastic OS cells and carcinoma-associated fibroblasts (*P*-value = 1). The *GDNF* gene was not DE between B cells and osteoblastic OS cells (*P*-value = 7.67 × 10^−1^).

It appeared that the *PSPN* gene exhibited no significant DE between osteoblastic OS cells and NK/T cells (*P*-value = 6.56 × 10^−7^). Moreover, the *PSPN* gene did not show DE between osteoblastic OS cells and myeloid cells 2 (*P*-value = 1) and osteoblastic OS cells and plasmocytes (1.65 × 10^−1^). Additionally, there was no significant DE of the *PSPN* gene between osteoblastic OS cells and osteoclasts (*P*-value = 2.27 × 10^−5^) and osteoblastic OS cells and endothelial cells (*P*-value = 6.75 × 10^−3^). Furthermore, the *PSPN* gene was not DE between osteoblastic OS cells and carcinoma-associated fibroblasts (*P*-value = 1). The *PSPN* gene was not DE between B cells and oteoblastic OS cells (*P*-value = 1).

**Figure 5.**
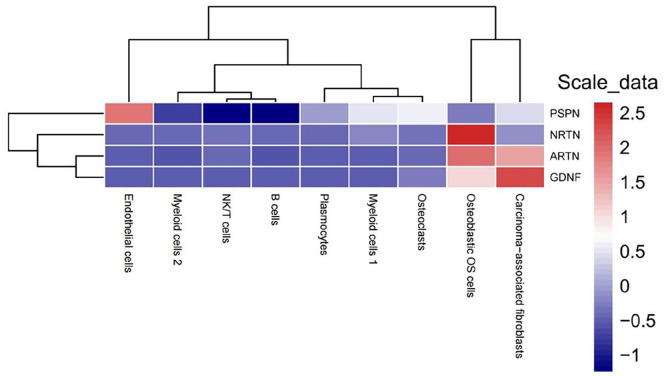
Heatmap displaying mRNA gene expression in TPM of four pain related genes *PSPN, NRTN, ARTN*, and *GDNF* in 9 OS cell types.

The *NRTN* gene had the highest expression in osteoblastic OS cells compared with all other OS cell types (Figures 4 and 5). It was revealed that the *NRTN* gene exhibited no significant DE between osteoblastic OS cells and NK/T cells (*P*-value = 1.88 × 10^−13^). Moreover, the *NRTN* gene did not show DE between osteoblastic OS cells and myeloid cells 2 (*P*-value = 5.20 × 10^7^) and osteoblastic OS cells and plasmocytes (1.79 × 10^−2^). Additionally, there was no significant DE of the *NRTN* gene between osteoblastic OS cells and osteoclasts (*P*-value = 7.84 × 10^−4^) and osteoblastic OS cells and endothelial cells (*P*-value = 2.25 × 10^−1^). Furthermore, the *NRTN* gene was not DE between osteoblastic OS cells and carcinoma-associated fibroblasts (*P*-value = 1). Finally, the *NRTN* gene was not DE between B cells and osteoblastic OS cells (*P*-value = 4.11 × 10^−1^).

### ARTN, GDNF, NRTN, and PSPN Gene Expression in Myeloid Cells 1 Versus All Other OS Cells

Our results showed that the *ARTN* gene exhibited significant DE between myeloid cells 1 and osteoblastic OS cells (*P*-value = 9.75 × 10^−42^). It was observed that the *ARTN* gene exhibited significant DE between myeloid cells 1 and NK/T cells (*P*-value = 1). However, the *ARTN* gene did not show DE between myeloid cells 1 and myeloid cells 2 (*P*-value = 1) and myeloid cells 1 and plasmocytes (*P*-value = 1). Additionally, there was no significant DE of the *ARTN* gene between myeloid cells 1 and osteoclasts (*P*-value = 1) and myeloid cells 1 and endothelial cells (*p*-value = 1). Furthermore, the *ARTN* gene was not DE between myeloid cells 1 and carcinoma-associated fibroblasts (*P*-value = 2.5 × 10^−15^). The *ARTN* gene was not DE between B cells and myeloid cells 1 (*P*-value 1).

Our results showed that the *GDNF* gene exhibited significant DE between myeloid cells 1 and osteoblastic OS cells (*P*-value = 7.76 × 10^−15^). It was observed that the *GDNF* gene exhibited significant DE between myeloid cells 1 and NK/T cells (*P*-value = 1.94 × 10^−1^). However, the *GDNF* gene did not show DE between myeloid cells 1 and myeloid cells 2 (*P*-value = 1) and myeloid cells 1 and plasmocytes (*P*-value = 1). Additionally, there was no significant DE of the *GDNF* gene between myeloid cells 1 and osteoclasts (*P*-value = 1.94 × 10^−1^) and myeloid cells 1 and endothelial cells (*P*-value = 1). Furthermore, the *GDNF* gene was not DE between myeloid cells 1 and carcinoma-associated fibroblasts (*P*-value = 1.5 × 10^−16^). The *GDNF* gene was not DE between B cells and myeloid cells 1 (*P*-value 1).

It was revealed that the *PSPN* gene exhibited no significant DE between myeloid cells 1 and osteoblastic OS cells (*P*-value = 3.94 × 10^−4^). We observed that the *GDNF* gene exhibited significant DE between myeloid cells 1 and NK/T cells (*P*-value = 3.12 × 10^−17^). However, the *GDNF* gene did not show DE between myeloid cells 1 and myeloid cells 2 (*P*-value = 2.83 ×10^−3^) and myeloid cells 1 and plasmocytes (*P*-value = 2.34 × 10^−3^). Additionally, there was no significant DE of the *GDNF* gene between myeloid cells 1 and osteoclasts (*P*-value = 7.611 × 10^−1^) and myeloid cells 1 and endothelial cells (*P*-value = 1). Furthermore, the *GDNF* gene was not DE between myeloid cells 1 and carcinoma-associated fibroblasts (*P*-value = 9.7 × 10^−1^). The *GDNF* gene was not DE between B cells and myeloid cells 1 (*P*-value = 2.6 × 10^−1^).

It appeared that the *NRTN* gene exhibited significant DE between myeloid cells 1 and osteoblastic OS cells (*P*-value = 2.38 × 10^−20^). It was observed that the *NRTN* gene exhibited no significant DE between myeloid cells 1 and NK/T cells (*P*-value = 1). However, the *NRTN* gene did not show DE between nyeloid cells 1 and myeloid cells 2 (*P*-value = 1) and myeloid cells 1 and plasmocytes (*P*-value = 1). Additionally, there was no significant DE of the *NRTN* gene between myeloid cells 1 and osteoclasts (*P*-value = 1) and myeloid cells 1 and endothelial cells (*P*-value = 1). Furthermore, the *NRTN* gene was not DE between myeloid cells 1 and carcinoma-associated fibroblasts (*P*-value = 1). The *NRTN* gene was not DE between B cells and myeloid cells 1 (*P*-value = 1).

### ARTN, GDNF, NRTN, and PSPN Gene Expression in Myeloid Cells 2 Versus Other OS Cells

Our results showed that the *ARTN* gene exhibited significant DE between myeloid cells 2 and osteoclasts (*P*-value = 8.21 × 10^−5^). It was observed that the *ARTN* gene exhibited significant DE between myeloid cells 2 and carcinoma-associated fibroblasts (*p*-value = 1). However, the *ARTN* gene did not show DE between plasmocytes and myeloid cells 2 (*P*-value = 4.15 × 10^−1^) and myeloid cells 2 and endothelial cells (*P*-value = 3.11 × 10^−3^). Additionally, there was no significant DE of the *ARTN* gene between myeloid cells 2 and B cells (*P*-value = 1).

Our results showed that the *GDNF* gene exhibited significant DE between myeloid cells 2 and osteoclasts (*P*-value = 7.73 × 10^−1^). It was observed that the *GDNF* gene exhibited significant DE between myeloid cells 2 and carcinoma-associated fibroblasts (*P*-value = 2.95 × 10^−5^).

Our results showed that the *PSPN* gene exhibited significant DE between myeloid cells 2 and osteoclasts (*P*-value = 8.21 × 10^−5^). It was observed that the *PSPN* gene exhibited significant DE between myeloid cells 2 and carcinoma-associated fibroblasts (*P*-value = 1). However, the PSPN gene did not show DE between plasmocytes and myeloid cells 2 (*P*-value = 4.15 × 10^−1^) and myeloid cells 2 and endothelial cells (*P*-value = 3.11 × 10^−3^). Additionally, there was no significant DE of the *PSPN* gene between myeloid cells 2 and B cells (*P*-value = 1).

It appeared that the *NRTN* gene exhibited no significant DE between myeloid cells 1 and osteoblastic OS cells (*P*-value = 1). It was observed that the *NRTN* gene exhibited no significant DE between myeloid cells 1 and NK/T cells (*P*-value = 1). However, the *NRTN* gene did not show DE between myeloid cells 1 and myeloid cells 2 (*P*-value = 1) and myeloid cells 1 and plasmocytes (*P*-value = 1). Additionally, there was no significant DE of the *NRTN* gene between myeloid cells 1 and osteoclasts (*P*-value = 1) and myeloid cells 1 and endothelial cells (*P*-value = 1). Furthermore, the *NRTN* gene was not DE between myeloid cells 1 and carcinoma-associated fibroblasts (*P*-value = 1). The *NRTN* gene was not DE between B cells and myeloid cells 1 (*P*-value = 1).

### ARTN, GDNF, NRTN, and PSPN Gene Expression in Osteoclasts Versus Other OS Cells

Our results showed that the *ARTN* gene exhibited no significant DE between plasmocytes and osteoclasts (*P*-value = 1). It was observed that the *ARTN* gene exhibited no significant DE between osteoclasts and endothelial cells (*P*-value = 1). The *ARTN* gene did not show DE between osteoclasts and B cells (*P*-value = 1). The *ARTN* gene was not DE between osteoclasts and carcinoma associated fibroblasts (*P*-value = 1.43 × 10^−2^).

Our results showed that the *GDNF* gene exhibited no significant DE between plasmocytes and osteoclasts (*P*-value = 1). It was observed that the *GDNF* gene exhibited no significant DE between osteoclasts and endothelial cells (*P*-value = 1). However, the *GDNF* gene did not show DE between osteoclasts and B cells (*P*-value = 1). The *GDNF* gene was not DE between osteoclasts and carcinoma associated fibroblasts (*P*-value = 4.81 × 10^−2^).

Our results showed that the *NRTN* gene exhibited no significant DE between plasmocytes and osteoclasts (*P*-value = 1). It was observed that the *NRTN* gene exhibited no significant DE between osteoclasts and endothelial cells (*P*-value = 1). However, the *NRTN* gene did not show DE between osteoclasts and B cells (*P*-value = 1). The *NRTN* gene was not DE between osteoclasts and carcinoma associated fibroblasts (*P*-value = 1.43 × 10^−2^).

Our results showed that the *PSPN* gene exhibited no significant DE between plasmocytes and osteoclasts (*P*-value = 1.059 × 10^−4^). We observed that the *PSPN* gene exhibited no significant DE between osteoclasts and endothelial cells (*P*-value = 1). However, the *PSPN* gene did not show DE between osteoclasts and B cells (*P*-value = 6.398 × 10^2^). The *PSPN* gene was not DE between osteoclasts and carcinoma associated fibroblasts (*P*-value = 1.61 × 10^−1^).

### ARTN, GDNF, NRTN, and PSPN Gene Expression in Carcinoma-Associated Fibroblasts Versus All Other OS Cells

The *GDNF* gene had the highest expression in carcinoma-associated fibroblasts compared with all other OS cell types (Figure 5). Our results showed that the *ARTN* gene exhibited no significant DE between plasmocytes and carcinoma associated fibroblasts (*P*-value = 8.183 × 10^−2^). It was observed that the *ARTN* gene exhibited no significant DE between carcinoma associated fibroblasts and endothelial cells (*P*-value = 3.76 × 10^−1^). The *ARTN* gene did not show DE between carcinoma associated fibroblasts and B cells (*P*-value = 2.29 × 10^−1^).

Our results showed that the *GDNF* gene exhibited no significant DE between plasmocytes and carcinoma associated fibroblasts (*P*-value = 8.12 × 10^−2^). It was observed that the *GDNF* gene exhibited no significant DE between carcinoma associated fibroblasts and endothelial cells (*P*-value = 4.11 × 10^−1^). the *GDNF* gene did not show DE between carcinoma associated fibroblasts and B cells (*P*-value = 2.29 × 10^−1^).

Our findings demonstrated that the *NRTN* gene exhibited no significant DE between plasmocytes and carcinoma associated fibroblasts (*P*-value = 1). It was observed that the *NRTN* gene exhibited no significant DE between carcinoma associated fibroblasts and endothelial cells (*P*-value = 1). The *NRTN* gene did not show DE between carcinoma associated fibroblasts and B cells (*P*-value = 1).

Our results showed that the *PSPN* gene exhibited no significant DE between plasmocytes and carcinoma associated fibroblasts (*P*-value = 1.611 ×10^−1^). We observed that the *PSPN* gene exhibited no significant DE between carcinoma associated fibroblasts and endothelial cells (*P*-value = 2.7 × 10^−1^). The *PSPN* gene did not show DE between carcinoma associated fibroblasts and B cells (*P*-value = 1).

### ARTN, GDNF, NRTN, and PSPN Gene Expression in Plasmocytes Versus Other OS Cells

Our results showed that the *ARTN* gene exhibited no significant DE between plasmocytes and endothelial cells (*P*-value = 1). It was observed that the *ARTN* gene exhibited no significant DE between plasmocytes and B cells (*P*-value = 1).

It was revealed that the *PSPN* gene exhibited no significant DE between plasmocytes and endothelial cells (*P*-value = 1.30 × 10^−4^). We observed that the *PSPN* gene exhibited no significant DE between plasmocytes and B cells (*P*-value = 1).

### ARTN, GDNF, NRTN, and PSPN Gene Expression in Endothelial Cells Versus All Other OS Cells

The *ARTN* gene had the highest expression in endothelial cells compared with all other OS cell types (Figure 5). Our results showed that the *ARTN* gene exhibited no significant DE between B cells and endothelial cells (*P*-value = 1). It was found that the *ARTN* gene exhibited no significant DE between B cells and endothelial cells (*P*-value = 6.4 × 10^−2^).

### Expression Level of ARTN, GDNF, PSPN, and NRTN Genes in 16 OS Cell Lines

**Figure 6.**
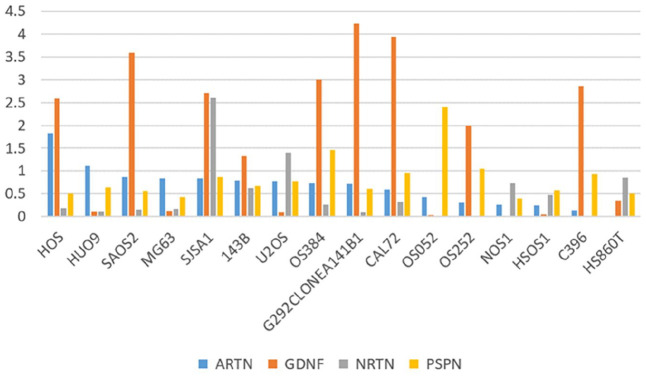
Genes expression in Cancer Cell Line Encyclopedia (CCLE) datasets. Bar plot displaying mRNA gene expression in TPM of four pain related genes (*PSPN, NRTN, ARTN*, and *GDNF*) in 16 OS cell lines.

OS has many subtypes, and different subtypes of OS display various histopathological patterns and clinical behaviour and would have different tumour microenvironment and gene expression profile.^1^ Using 16 Cancer Cell Line Encyclopedia (CCLE) datasets of OS cell lines, we showed varying expression levels for the four pain-associated genes (*ARTN, GDNF, NRTN, and PSPN*; Figure 6). Specifically, the *GDNF* gene had the highest expression level of 4.24 in the G292CLONEA141B1 cell line (Figure 6). The *NRTN* gene was most highly expressed at 2.6 in the SISA1 cell line. The *ARTN* gene reached its peak expression of 1.8 in the HOS cell line. Lastly, the *PSPN* gene had its highest expression of 2.4 in the OS052 cell line (Figure 6).

### Survival Analysis of ARTN, GDNF, PSPN, and NRTN Gene Expression in OS

**Figure 7.**
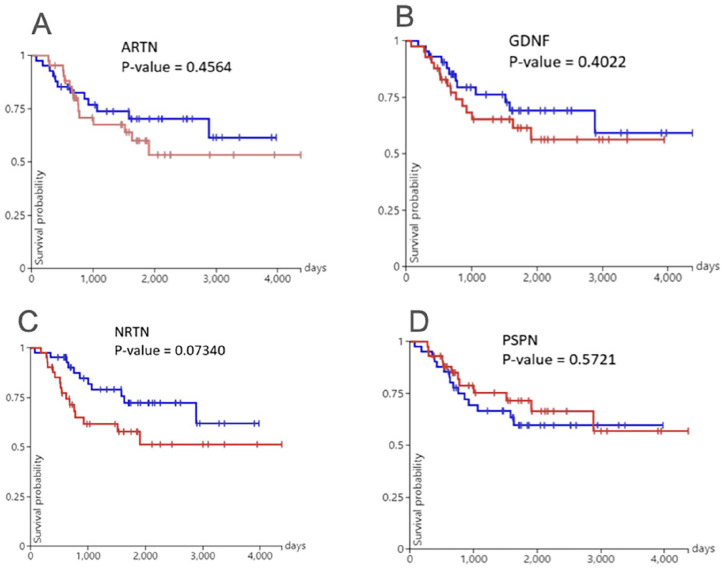
Plots of Kaplan-Meier analysis of the survival rate of OS patients with the expression levels of *ARTN* (A), *GDNF* (B), *NRTN* (C), and *PSPN* (D) genes. Red colour represents genes with high expression, whereas blue colour represents genes with low expression. No significant difference in survival probability was found between high and low expression of these genes in OS patients.

We then compared survival in OS patients based on the four genes. Our results showed that all four genes were not statistically significant in their association with overall OS patient survival (Figure 7A-D). We found OS patients with lowest survival expressing the *NRTN* gene (Figure 7C). The expression of *PSPN* in OS patients showed overall highest survival (Figure 7D).

## Discussion

OS is rare and aggressive bone tumour that is most common in children and young adults. Our understanding of the OS tumour microenvironment is lacking and treatment for the management of OS pain is an unmet clinical need.^20^ In this study, we have measured and compared the gene expression level of four pain related genes in diverse OS cells using scRNA seq bioinformatic analyses. Myeloid cells 1 is the main cell type found in the OS tumour microenvironment followed by osteoblastic OS cells, NK/T cells, myeloid cells 2, osteoclasts, carcinoma associated fibroblasts, plasmocytes, endothelial cells, and B cells, respectively. Notably, we observed that the *ARTN* gene had the highest expression in osteoblastic OS cells (Figure 4). The *GDNF* gene had the highest expression in carcinoma associated fibroblasts (Figure 4). The *NRTN* gene also had the highest expression in osteoblastic OS cells (Figure 4). *PSPN* gene exhibited the highest expression in endothelial cells (Figure 4).

The *ARTN* gene, also known as artemin, is a member of GDNF family of ligands.^14^ This gene encodes a protein that plays a significant role in neurodevelopment and various cellular processes.^14^ In cancer, *ARTN* has been shown to promote tumour growth, metastases, and drug resistance.^14^ Our study showed that the ART gene was highly expressed in osteoblastic OS cells in OS tumours (Figure 4), suggesting that *ARTN* gene expression in osteoblastic OS cells might be involved in OS pain.

*GDNF* is a neurotrophic factor traditionally known for its role in the development and maintenance of the nervous system.^21,22^ Recent research has expanded its relevance to oncology, particularly in OS.^23^ In the context of OS, *GDNF* has an emerging role in tumour growth, treatment resistance, and pain.^13,22,24^ Studies have shown that *GDNF* can influence OS cell proliferation, survival, and metastasis. The interaction between *GDNF* and its receptor, GFRα1, may affect tumour cell behaviour and contribute to the aggressive nature of OS.^24^ This signalling pathway has been linked to increased tumour cell viability and resistance to apoptosis, which can exacerbate the disease. Furthermore, *GDNF*’s role in OS suggests potential therapeutic strategies. Targeting the GDNF/GFRα1 signalling axis could offer new avenues for treatment, aiming to inhibit tumour growth and improve patient outcomes. In the present study, we have found that *GDNF* gene was highly expressed in carcinoma associated fibroblasts among a diverse range of OS cells. Understanding the mechanistic involvement of *GDNF* in OS could provide valuable insights into developing novel targeted therapies and enhancing current treatment regimens.

The *NRTN* gene encodes neurturin that is closely related to GDNF.^13^ Neurturin, like *GDNF*, plays a significant role in neuronal survival and development.^25^ Emerging research has highlighted its relevance beyond neurobiology, particularly in oncology, where its expression, and function are being investigated in OS. In OS, *NRTN* has been found to influence tumour behaviour, progression and pain.^13^ Studies suggest that *NRTN* can impact OS cell proliferation, migration, and survival, potentially contributing to the aggressive nature of the disease. The *NRTN* gene’s product exerts its effects through interaction with the GFRα2 receptor and the Ret tyrosine kinase, pathways that are also implicated in the pathology of OS.^24^ The involvement of *NRTN* in OS indicates its potential as a biomarker for tumour progression and a target for therapeutic intervention.^24^ By modulating the NRTN/GFRα2/Ret signalling axis, it might be possible to develop novel strategies to inhibit tumour growth and improve treatment outcomes for patients with OS.^24^ Understanding the role of the *NRTN* gene in OS provides important insights into the tumour’s molecular mechanisms and opens avenues for targeted therapies aimed at disrupting its growth and spread.

The *PSPN* gene encodes a protein that is involved in various cellular processes including growth, survival, and differentiation.^24,26^ In the context of OS, *PSPN* has emerged as a significant factor influencing tumour behaviour and pain.^13^ Research has shown that *PSPN* is often overexpressed in OS tissues compared to normal bone tissues. This overexpression is associated with increased tumour cell proliferation, enhanced invasion, and poor clinical outcomes.^24^ The role of *PSPN* in OS suggests that it may act as a potential biomarker for diagnosis and prognosis. Additionally, targeting *PSPN* or its associated pathways could offer new therapeutic strategies for combating OS induced pain. In our research, we showed that *PSPN* was not significantly DE between the different OS cells. In addition, *PSPN* gene was highly expressed in endothelial cells and lowly expressed in NK/T cells and B cells (Figure 4), suggesting its potential role in the angiogenesis of OS.

Previous findings demonstrate osteoclast-induced bone destruction plays a role in bone cancer pain.^27^ The tumour can also invade or compress nearby peripheral nerves, causing pain through direct nerve involvement.^28^ In cases of metastatic disease, especially when the tumour spreads to internal organs (eg, lungs), visceral pain may also develop.^29^ Further research is required to determine the precise mechanism of action related to neuropathic pain, such as rearrangement during transfection (RET) tyrosine kinase receptor signalling pathway.^30,31^

## Conclusion

In summary, this paper investigates the expression of four pain related genes in OS tissues and OS cell lines, and predict their role in associate with OS pain and survival. Our results show that the panel of four genes, *PSPN, NRTN, GDNF*, and *ARTN* genes are DE between some OS cells, however a majority of the genes were not DE in these cells. *ARTN* and *NRTN* genes are expressed most abundantly in osteoblastic OS cells, *GDNF* in carcinoma associated fibroblasts, and *PSPN* in endothelial cells. Further research into mechanistic insights into pain-related genes is crucial for improving the quality of life of OS patients in order to manage severe pain and suffering experienced during disease progression. For instance, preclinical studies using human OS cell lines to inhibit the pain genes in mice would be employed to confirm their effects in vivo, thus paving the way for therapeutic development of OS using these targets.

## References

[1] Rothzerg E Xu J Wood D. Different subtypes of osteosarcoma: histopathological patterns and clinical behaviour. J Mol Pathol. 2023;4(2):99-108.

[2] Prater S McKeon B. Osteosarcoma. Treasure Island (FL): StatPearls Publishing. 2023. 31751058

[3] Haque A Engel J Teichmann SA Lönnberg T. A practical guide to single-cell RNA-sequencing for biomedical research and clinical applications. Genome Med. 2017;9(1):75-12. 10.1186/s13073-017-0467-4 PMC5561556 28821273

[4] Feleke M Feng W Song D , et al. Single-cell RNA sequencing reveals differential expression of EGFL7 and VEGF in giant-cell tumor of bone and osteosarcoma. Exp Biol Med. 2022;247(14):1214-1227. 10.1177/15353702221088238 PMC9379604 35695550

[5] Feleke M Feng W Rothzerg E , et al. Single-cell RNA-seq identification of four differentially expressed survival-related genes by a TARGET: osteosarcoma database analysis. Exp Biol Med. 2022;247(11):921-930. 10.1177/15353702221080131 PMC9189571 35285281

[6] Chen J Yan C Yu H Zhen S Yuan Q. miR-548d-3p inhibits osteosarcoma by downregulating KRAS. Aging. 2019;11(14):5058-5069. 31327761 10.18632/aging.102097 PMC6682526

[bibr7-11769351251331508] Saba KH Difilippo V Kovac M , et al. Disruption of the TP53 locus in osteosarcoma leads to TP53 promoter gene fusions and restoration of parts of the TP53 signalling pathway. J Pathol. 2024;262(2):147-160. 38010733 10.1002/path.6219

[8] Czarnecka AM Synoradzki K Firlej W , et al. Molecular biology of osteosarcoma. Cancers. 2020;12(8):2130. 32751922 10.3390/cancers12082130 PMC7463657

[9] Parvizi J. High Yield Orthopaedics E-book. Elsevier Health Sciences; 2010.

[10] Cleeland CS. The measurement of pain from metastatic bone disease: capturing the patient’s experience. Clin Cancer Res. 2006;12(20):6236s-6242S. 10.1158/1078-0432.CCR-06-0988 17062707

[11] Ding Z Xu W Zhang J , et al. Normalizing GDNF expression in the spinal cord alleviates cutaneous hyperalgesia but not ongoing pain in a rat model of bone cancer pain. Int J Cancer. 2017;140(2):411-422. 27716965 10.1002/ijc.30438

[12] Vieira C Fragoso M Pereira D Medeiros R. Pain prevalence and treatment in patients with metastatic bone disease. Oncol Lett. 2019;17(3):3362-3370. 30867771 10.3892/ol.2019.10013 PMC6396205

[13] Nencini S Ringuet M Kim D-H Greenhill C Ivanusic JJ. GDNF, neurturin, and artemin activate and sensitize bone afferent neurons and contribute to inflammatory bone pain. ARC J Neurosci. 2018;38(21):4899-4911. 10.1523/JNEUROSCI.0421-18.2018 PMC6596122 29712778

[14] Zhu S Li Y Bennett S , et al. The role of glial cell line-derived neurotrophic factor family member artemin in neurological disorders and cancers. Cell Prolif. 2020;53(7):e12860. 10.1111/cpr.12860 PMC7377943 32573073

[15] Kuijjer ML Peterse EF van den Akker BE , et al. IR/IGF1R signaling as potential target for treatment of high-grade osteosarcoma. BMC Cancer. 2013;13(1):9. 23688189 10.1186/1471-2407-13-245 PMC3672007

[16] Kuijjer ML Rydbeck H Kresse SH , et al. Identification of osteosarcoma driver genes by integrative analysis of copy number and gene expression data. Genes Chromosomes Cancer. 2012;51(7):696-706. 22454324 10.1002/gcc.21956

[17] Feng W Lin H Rothzerg E , et al. RNA-seq and single-cell transcriptome analyses of TRAIL receptors gene expression in human osteosarcoma cells and tissues. Cancer Inform. 2023;22:11769351231161478. 10.1177/11769351231161478 PMC10123892 37101729

[18] Liu Y Feng W Dai Y , et al. Single-cell transcriptomics rev-eals the complexity of the tumor microenvironment of treatment-naive osteosarcoma. Front Oncol. 2021;11:709210. 34367994 10.3389/fonc.2021.709210 PMC8335545

[19] Barretina J Caponigro G Stransky N , et al. The Cancer Cell Line Encyclopedia enables predictive modelling of anticancer drug sensitivity. Nature. 2012;483(7738):603-607. 22460905 10.1038/nature11003 PMC3320027

[20] Bencze N Scheich B Szőke , et al. Osteosarcoma-induced pain is mediated by glial cell activation in the spinal dorsal horn, but not capsaicin-sensitive nociceptive neurons: A complex functional and morphological characterization in mice. Cancers. 2024;16(10):1788. 38791867 10.3390/cancers16101788 PMC11120600

[21] Arango-Lievano M Jeanneteau F . Timing and crosstalk of glucocorticoid signaling with cytokines, neurotransmitters and growth factors. Pharmacol Res. 2016;113(Pt A):1-17. 10.1016/j.phrs.2016.08.005 27498156

[22] Nguyen TM Ngoc DTM Choi J-H Lee C-H. Unveiling the neural environment in cancer: exploring the role of neural circuit players and potential therapeutic strategies. Cells. 2023;12(15):1996. 37566075 10.3390/cells12151996 PMC10417274

[23] Fielder GC Yang TW-S Razdan M , et al. The GDNF family: a role in cancer? Neoplasia. 2018;20(1):99-117. 29245123 10.1016/j.neo.2017.10.010 PMC5730419

[24] Li Q Cao Z Zhao S. The emerging portrait of glial cell line-derived neurotrophic factor family receptor alpha (GFRα) in cancers. Int J Med Sci. 2022;19(4):659-668. 35582425 10.7150/ijms.64133 PMC9108399

[25] Sullivan AM Toulouse A. Neurotrophic factors for the treatment of Parkinson’s disease. Cytokine Growth Factor Rev. 2011;22(3):157-165. 21689963 10.1016/j.cytogfr.2011.05.001

[26] Yang J Lindahl M Lindholm P , et al. PSPN/GFRα4 has a significantly weaker capacity than GDNF/GFRα1 to recruit RET to rafts, but promotes neuronal survival and neurite outgrowth. FEBS Lett. 2004;569(1-3):267-271. 15225646 10.1016/j.febslet.2004.06.007

[27] Jimenez-Andrade JM Mantyh WG Bloom AP , et al. Bone cancer pain. Ann N Y Acad Sci. 2010;1198:173-181. 20536932 10.1111/j.1749-6632.2009.05429.x PMC5642911

[28] Yoon SY Oh J. Neuropathic cancer pain: prevalence, pathophysiology, and management. Korean J Intern Med. 2018;33(6):1058-1069. 29929349 10.3904/kjim.2018.162 PMC6234399

[29] Ahmad I Ahmed MM Ahsraf MF , et al. Pain management in metastatic bone disease: a literature review. Cureus. 2018;10(9):10. 10.7759/cureus.3286 PMC6235631 30443456

[30] Mahato AK Sidorova YA. Glial cell line-derived neurotrophic factors (GFLs) and small molecules targeting RET receptor for the treatment of pain and Parkinson’s disease. Cell Tissue Res. 2020;382(1):147-160. 32556722 10.1007/s00441-020-03227-4 PMC7529621

[31] Mahato AK Sidorova YA. RET receptor tyrosine kinase: role in neurodegeneration, obesity, and cancer. Int J Mol Sci. 2020;21(19):7108. 32993133 10.3390/ijms21197108 PMC7583994

